# Retraction: “A Dynamic Adaptive Ensemble Learning Framework for Noninvasive Mild Cognitive Impairment Detection: Development and Validation Study”

**DOI:** 10.2196/77635

**Published:** 2025-05-23

**Authors:** JMIR Editorial Office

**Affiliations:** 1 JMIR Publications, 130 Queens Quay E, Suite 1100, Toronto, ON, M5A 0P6, Canada, 1 416 583 2040

The JMIR Editorial Office is retracting the article “A Dynamic Adaptive Ensemble Learning Framework for Noninvasive Mild Cognitive Impairment Detection: Development and Validation Study” by Li et al [1] following investigation due to concerns over irregularities during the peer review process. We regret that these issues were not resolved prior to publication.

All authors disagreed with the retraction decision.
